# Sepsis and Nosocomial Infections: The Role of Medico-Legal Experts in Italy

**DOI:** 10.3390/antibiotics8040199

**Published:** 2019-10-28

**Authors:** Lucia Tattoli, Alessandro Dell’Erba, Davide Ferorelli, Annarita Gasbarro, Biagio Solarino

**Affiliations:** 1 S.C. Medicina Legale U - Azienda Ospedaliero-Universitaria Città della Salute e della Scienza di Torino, 10126 Torino, Italy; 2 Institute of Legal Medicine Department of Medicine (DIM), School of Medicine, University of Bari A. Moro, 70124 Bari, Italy; adellerba@yahoo.com (A.D.); davideferorelli@gmail.com (D.F.); biagiosolarino@libero.it (B.S.); 3 Pharmacy University Hospital Policlinico, 70124 Bari, Italy; anna.gasbarro@policlinico.ba.it

**Keywords:** sepsis, septic shock, inflammation, nosocomial infection, risk management, forensic, postmortem diagnosis, malpractice, medico-legal expert

## Abstract

Sepsis is a leading cause of morbidity and mortality worldwide. It is defined as the presence of a Systemic Inflammatory Response Syndrome, and it represents a significant burden for the healthcare system. This is particularly true when it is diagnosed in the setting of nosocomial infections, which are usually a matter of concern with regard to medical liability being correlated with increasing economic costs and people’s loss of trust in healthcare. Hence, the Italian governance promotes the clinical risk management with the aim of improving the quality and safety of healthcare services. In this context, the role of medico-legal experts working in a hospital setting is fundamental for performing autopsy to diagnose sepsis and link it with possible nosocomial infections. On the other hand, medico-legal experts are party to the clinical risk management assessment, and deal with malpractice cases and therefore contribute to formulating clinical guidelines and procedures for improving patient safety and healthcare providers’ work practices. Due to this scenario, the authors here discuss the role of medico-legal experts in Italy, focusing on sepsis and nosocomial infections.

## 1. Introduction

Sepsis is a leading cause of morbidity and mortality worldwide. It refers to a syndrome of physiologic, pathological, and biochemical abnormalities induced by infection. Despite the efforts of the scientific community to reevaluate and revise clinical, imaging, and laboratory features, the diagnosis of sepsis is still challenging [1]. In this ongoing research, further understanding of the biology of sepsis, the availability of new diagnostic approaches, and enhanced collection of data will be helpful for clinicians to standardize their approach in these cases [2]. 

Nosocomial infections (NI) are defined as infections acquired in hospitals by patients admitted for a reason other than that infection. The infection is not present or incubating at the time of admission and can appear after discharge. The most frequent NI are those resulting from surgical wounds, urinary tract infections, and lower respiratory tract infections [3]. Hospital-Acquired Infections (HAI) constitute a burden on the healthcare system in terms of high economic costs and patients’/caregivers’ loss of trust. Claims concerning NI advanced by patients against public or private hospitals are a matter of great concern in Italy [4].

In recent decades, the Italian governance has promoted Clinical Risk Management (CRM) with the aim of improving the quality and safety of healthcare services. On the other hand, a new normative scenario has been established, whereby inpatient institutions have the possibility of directly compensating damage claims arising from alleged medical negligence, thus obviating the cost of liability insurance. In this setting, a fundamental role is reserved for medico-legal experts. The four years training in forensic (legal) medicine offered by Italian universities concentrates on general practices in forensic pathology, including autopsy and the scientific methods applied in crime investigation. In addition, physicians are trained on evaluation of damages in personal injury cases, including those linked with medical malpractice [5].

Hence, hospital medico-legal experts perform medical autopsy and are on the staff of the Risk Management Unit (RMU) that evaluate the malpractice claims, compensate damages, and lay down elaborate care pathways for preventing errors.

The association between sepsis and medical autopsy is not new in medical literature, as evidenced by the Latin axiom mortui vivos docent (‘The dead teach the living’). Ignaz Philipp Semmelweis was considered the father and pioneer of improved NI prevention, thanks to his job in supervised medical autopsy [6]. The diagnosis of sepsis and NI is a challenge not only for clinicians but also for forensic pathologists, because of the difficulty of histological and microbiological postmortem examinations. Hence, the evidence of NI needs to be compared with hospital use of best practice infection control measures. Only a correct evaluation of all these elements will allow identifying and adequately preventing adverse events and errors, thereby improving healthcare system.

## 2. Postmortem Diagnosis of Infection-Related Fatalities

Diagnosis of sepsis deserves more attention in hospital settings when hypothesis of malpractice arises after the death—often unexpected—of inpatients. Sepsis has mortality rates as high as 30–90% [7], and it is a common cause of severe illness in patients with community-acquired and NI [8]. Deaths can be associated with infection due to a medical surgical device (e.g., vascular catheters, tracheostomy tube, drainage, etc.), long-lasting and uncritical administration of broad-spectrum antibiotics, nursing neglect, infected surgical wounds, decubitus ulcers, infection of prosthetic material, etc. [7]. 

When the forensic pathologist manages a sepsis fatality, the questions should be:

- Did the patient suffer from a septic condition prior to death?
- Did sepsis cause or contribute to death?
- What was the specific portal of entry of microorganisms?
- Have clinical diagnosis and treatment been prompt and accurate?
- Could the patient’s death have been avoided with different management?

The aim of autopsy and the following investigations is to understand if other underlying diseases could have been overlooked, contributing to sepsis and fatal outcome in sepsis-related fatalities of inpatients. The postmortem diagnosis of sepsis is by far the most difficult to establish in forensic pathology. 

Sepsis is characterized by life-threatening organ dysfunctions due to dysregulation of the host’s immune and metabolic response to infection [7]. Innate immunity involving cells (macrophages, neutrophils, dendritic, and natural killer cells) and humoral factors (complement, antibodies, and acute phase proteins) is activated by pathogen entry, and the infection is eradicated. Nevertheless, these responses may cause tissue injury and multiple organ dysfunction syndrome if dysregulated, as occurs during sepsis [9,10]. A variety of life-threatening clinical conditions, including trauma, burns, extensive surgical procedures, protracted hemorrhagic, cardiac shock, etc., can provoke a similar systemic inflammatory reaction in the absence of infection, especially in the early stages [1,3]. The American College of Chest Physicians and the Society of Critical Care Medicine Consensus Conference (Northbrook, IL, USA; August 1991) recommended the term Systemic Inflammatory Response Syndrome (SIRS) to describe this inflammatory process which can result from either non-infectious or infectious conditions. SIRS is clinically defined by two or more specific clinical criteria which, when caused by infection, characterize “sepsis”. The host’s inflammatory response to infection progresses to sepsis, severe sepsis, and septic shock causing organ dysfunction syndrome and multiple organ failure [7,9]. Early diagnosis and management of sepsis are one of the greatest challenges of modern medicine worldwide, because no clinical gold standard is available, and the current analyses are not sensitive enough for use as reference standards. This difficulty also affects investigations in the forensic field, where a multidisciplinary approach including macroscopic, histological, microbiological, biochemical, and immunohistochemical investigations is mandatory, because clinical and circumstantial antemortem can often be lacking. Nevertheless, these investigations must follow sufficiently rigorous scientific criteria through the identification of reliable markers of sepsis.

At present, a forensic protocol or algorithm of action for postmortem diagnosis of infection-related fatalities has not been developed, even though the research about markers for sepsis has given promising results [11]. 

### 2.1. Circumstances of Death and Medical History

The first issue is examination of the circumstances of death and the medical history, especially the results from antemortem diagnostic procedures such as laboratory and microbiological tests [11]. Sometimes, a correct diagnosis of infection during the patient’s life cannot be established because the disease has an atypical, insidious, or even fulminant course. The acquisition and reexamination of specimens such as blood cultures taken before death is recommended prior to performing the autopsy, because it can be helpful for further diagnostic procedures, especially microbiological tests [9,11].

### 2.2. Collection of Specimens

During autopsy, blood samples for the determination of biochemical sepsis markers should be collected by aspiration with a sterile needle and syringe from the femoral vein, after the dissection of the upper thigh. At least two postmortem blood samples at different time points are recommended, with the aim of following the concentration of the parameters from the time of death [1]. Alternative matrices including urine, vitreous, pericardial, pleural, and cerebrospinal fluids have been evaluated when blood is unavailable at autopsy [8,12,13]. Microbiological investigations by conventional cultures in bacterial infections are still more common than DNA technology, because of their easy access and high cost-effectiveness [9]. Nevertheless, several parameters may influence and limit the diagnostic utility of postmortem cultures, such as sampling procedures and selection of suitable specimens for culture (swabs or blood), the time elapsed between death and the sampling with possible bacterial contamination, agonal spread of bacteria in non-septic subjects, or antibiotic therapy prior to death [9,13]. It is a fact that polymicrobial growth should be considered contamination in the majority of cases [9].

### 2.3. Biomarkers for Sepsis-Related Deaths

Several biomarkers have been evaluated for diagnosis of sepsis-related deaths, including biochemical molecules involved in the pathogenesis of sepsis cascade [7,8].

C reactive protein (CRP) is an inflammation marker of acute-phase response in infections (bacterial, viral, fungal), inflammatory diseases, necrosis (myocardial infarction, pancreatitis), trauma, and neoplasms [7,8]. Postmortem elevated CRP levels in serum and pericardial fluid have been evaluated in pre-autopsy screening for sepsis or ketoacidosis [14].

Procalcitonin (PCT) has an important role in the response to infections microbial infections [8,15]. Postmortem PCT levels have been investigated in blood, vitreous, pericardial, and cerebrospinal fluids, also by rapid diagnostic test, offering a useful marker in routine autopsy investigations for classifying death due to sepsis [7,8,16,17,18,19].

Lipopolysaccharide-binding protein (LPB) is released into blood as a type I acute-phase reactant after bacteremia or endotoxemia; an increase in postmortem serum LBP levels has been observed in sepsis groups, with a marked decrease correlated with interval after death [7,20].

Soluble interleukin-2 receptor (sIL-2R) is widely expressed by leukocytes as activated B cells, monocytes, eosinophil granulocytes, and natural killer cells. Its release in blood is an indirect marker of activation of T-cells which secrete IL-2 during sepsis [7,8,21]. 

Interleukin-6 (IL-6) is a proinflammatory cytokine expressed by leukocytes, fibroblasts, and endothelial cells in the early phase of inflammation [1,2]. Tsokos et al. found an increase of IL-6 levels in the postmortem serum of septic patients, with this increase correlating well with interval after death. Trauma or burn injury prior to death may increase IL-6 levels, and therefore the measurement of at least two postmortem CRP values seems to be warranted in order to identify sepsis-related fatalities when no anamnesis is available [22].

Postmortem course of Interleukin-1β (IL-1β), sIL-2R, and LBP has been evaluated in septic and non-septic fatalities, showing that sIL-2R and LBP can be useful in diagnosis of sepsis in forensic autopsy practice [22].

Endocan (endothelial cell-specific molecule-1) is a marker of endothelial activation by proinflammatory cytokines and proangiocenic molecules in septic cases; an increase of both endocan and PCT serum levels was found in septic cases [23].

Soluble CD14 subtype (sCD14-ST) presepsin is a type II acute-phase reactant and high-affinity receptor for LPS–LBP complexes in inflammation [8]. Increased postmortem serum sCD14-ST levels have been found in sepsis-related deaths, even though combined determination with PCT was proposed to improve their isolated diagnostic value [24].

The levels of copeptin were found to be significantly higher in sepsis cases, and the concentrations correlated with PCT, CRP, and IL-6 values [25]. 

Soluble triggering receptor expressed on myeloid cells-1 (sTREM-1) is an immunoglobulin expressed on monocyte and macrophage surfaces and upregulated in biological fluids and tissues infected by gram-positive and gram-negative bacteria [8]. Increased sTREM-1 levels were found in postmortem serum, pericardial fluid, and urine of septic death, cases, even though combined determination with PCT seems to improve the diagnostic value [26].

Postmortem serum levels of Troponin I, Troponin T, and NT-proBNP increased in sepsis related deaths in absence of any relevant cardiovascular disease [27].

Neopterin is a marker of T-cell activation and seems to be associated with SIRS; postmortem serum neopterin was investigated in bacterial and viral infection-related cases as well as in delayed deaths due to trauma [8,28]. A concurrent analysis of neopterin and CRP levels can be useful in the diagnosis of these infections [29].

Growth arrest-specific 6 (Gas6) is expressed in leukocytes, platelets, endothelial cells, and monocytes, and increased serum levels have been described in patients with sepsis and septic shock; it is considered a general marker of inflammation rather than a specific biomarker of sepsis [24].

According to the literature, the most useful postmortem marker of sepsis-related deaths may be serum PCT, CRP, sTREM-1, and sIL-2R [13]. Serial monitoring of serum CRP, PCT, IL-6, IL-1b, sIL-2R, and LBP after death has been suggested to be helpful for the postmortem diagnosis of sepsis, but more studies are needed [22,30].

### 2.4. Immunohistochemistry

The tracing of immunohistochemical antigens has been evaluated in the lungs and the liver as a sign of sepsis [8,31]. The findings are generally related to the systemic hypoperfusion damage, acute respiratory distress syndrome (ARDS), disseminated intravascular coagulation syndrome (DICS), organ dysfunction syndrome, and multiple organ failure with signs of myocardial ischemia, pulmonary and cerebral edema, spleen infarction, and often intestinal hemorrhages [7,8,9,32]. The lung is the primary target organ in sepsis. Therefore, the immunohistochemical detection of different markers of the inflammatory cellular response of the lungs has been investigated [1]. The most important postmortem markers include E-selectin, very late activation antigen-4 (VLA-4), vascular cell adhesion molecule-1 (VCAM-1), Intercellular adhesion molecule-1 (ICAM-1), Lactoferrin (LF), vascular endothelial growth factor (VEGF), angiostatin, vitreonectin, VE-cadherin, angiotensin-I-converting enzyme (ACE), and TNF-α (tumor necrosis factor α) [7,32]. According to the literature, PCT seems to be the most interesting marker of sepsis cases, even though the use of at least two to three markers contextually is suggested to increase the sensitivity and specificity of the molecules [32,33,34]. Postmortem alteration due to autolysis and putrefaction can interfere with the quality of immunostaining. Several factors regarding the cadaver (type of death, constitution) and the environment (temperature, ventilation, etc.) may affect the progress of putrefaction; therefore, conducting the autopsy immediately after death is recommended, even though the circumstances and legislation related to death may not permit it [32]. 

Moreover, there is a lack of immunohistochemical studies in sepsis, some of them having been conducted only on animals, and therefore it is difficult to use these methods in forensic practice at present.

### 2.5. Autopsy Findings

The absence of pathognomonic alteration of organs in sepsis-related deaths is an obstacle for the pathologist [9]. Postmortem macroscopic and microscopic findings reflect the systemic activation of pathogen-mediated inflammatory cascade producing cell and tissue injury that is neither specific nor sensitive for sepsis [32]. Therefore, the findings of potentially sepsis-induced alterations should be considered together with the entire case history and laboratory results.

External examination of the cadaver may reveal signs of infection such as inflammatory infiltrations, abscesses, infected wounds (burns), hemorrhagic lesions from DICS (as in meningococcal sepsi), viral/bacterial/fungal–related skin lesions, medical devices as a potential source of infection, and the degree of putrefaction despite the refrigeration of the body [7,9,11]. 

The internal examination may reveal [7,9,11,13,32]:

- Hemorrhagic lesions of pleural, pericardial, and peritoneal membranes as well as of the internal organs;
- Blood extravasations into the adrenal glands in Waterhouse–Friderichsen syndrome typical in meningococcal septicemia [35];
- Alteration of the lungs which are heavy, congested, edematous, producing frothy/bloody/purulent discharge;
- The heart with signs of endocarditis, necrosis, parietal thrombi, subepicardial hemorrhages, subendocardial hemorrhages (“Sheehan hemorrhages”), dilated and flaccid left ventricle for severe left ventricular dysfunction;
- Enlarged kidneys for cortical edema, congested medulla, septicopyemic abscesses, pyelonephritis, or ischemic necrosis of the renal cortex;
- Enlarged liver with a tense Glisson’s capsule and rounded edges because of accumulation of leukocytes and interstitial edema, signs of cholangitis (cholestatic jaundice);
- Enlarged and swollen spleen with septic thrombophlebitis or septicopyemic abscesses; the term acute splenitis (“septic spleen”) refers to a soft consistency of the pulp draining from cut sections;
- Cerebral infarction, distributed, round to conelike hemorrhages;
- Gastrointestinal shock lesions such as subserous petechial hemorrhages, erosions, and acute ulcers.

### 2.6. Histological Examination

Histopathological lesions may vary considerably, and inflammatory infiltration may demonstrate different degrees of severity. Involvement of the lungs as “shock lung” (alveolar damage secondary to ARDS, evidence of broncho-pneumonia, septic emboli in the vascular system), of the heart (interstitial infiltrations of mononucleated cells, microcirculatory fibrin deposits, necrosis, interstitial myocarditis, infectious endocarditis), of the liver (cholestasis, proliferation of biliary ducts, interstitial inflammatory infiltrations, aggregation of fibrin), of the spleen (acute hyperplasia of the red pulp and infiltrations of neutrophils and macrophages), of the brain (circumscribed loss of neurons in the hippocampus formation, proliferation of astrocytes, and microglia in the cerebral cortex), of the gastrointestinal tract, and of the adrenals should be evaluated [7,9,13,29]. Cadaveric bone marrow has been considered as an alternative matrix in postmortem investigations of sepsis, because of its good resistance to autolysis and contamination. The most common finding in biopsies was a prevalence of neutrophils and their precursors with increased cellularity and a large number of myeloid precursors [36]. 

In conclusion, several factors such as nutritional status, immunodeficiency, immunosuppression, and preexisting pathology should be taken into account when the pathologist faces a potential sepsis-related death. The course of the infectious disease may be influenced by these factors. Therefore, all of the findings at external examination of a deceased, as well as at autopsy, histology, biomarkers analysis, and immunohistochemistry must be interpreted with considerable caution [9]. 

A number of deaths of inpatients are investigated to establish the causal relationship between exogenous noxae and infection, and eventually death, because litigation for medical malpractice can arise. Despite advances in infection control measures, the diffusion of antibiotic resistance, the often delayed detection of microorganisms, and the difficult management of septicemia contribute to sepsis-related fatalities in hospital facilities. For example, infection is a leading cause of morbidity and mortality in burn patients [37]. Thermal injuries facilitate invasion by microorganisms determining immunodepression, severe catabolism, prolonged hospitalization, and invasive diagnostic and therapeutic procedures (e.g., endotracheal tubes, intravascular catheters, urinary catheters, etc.), all factors contributing to sepsis [38]. The infection’s portal of entry is often difficult to detect; there is also the issue of whether infections are absolutely avoidable, with regard to alleged medical malpractice. 

## 3. Patient Safety and Medical Liability in Italy

Patient safety and preventing medical liability are two of the most important issues in healthcare system politics in Italy. Dealing with the European Union recommendations and the initiatives conducted by several countries worldwide with the aim of decreasing the risk of harm associated with healthcare, the Italian government has promoted a program to develop activities related to patient safety and CRM since 2007 [39]. If to err is human, the physician’s apology must convey respect, mutual suffering, and responsibility [40,41]. 

In accordance with the evidence that errors are caused by system failures and not by bad doctors, the Italian Ministry of Health has generated a National Sentinel Event Reporting System, instituted hospital RMU, and implemented recommendations to prevent error and try to develop through training, education, and proactive processes a deep cultural change to improve the safety of the patient and operators [42,43,44]. This “new” healthcare discipline was mainly adopted due to increasing costs of litigation, directly involving medico-legal and insurance issues. It is well known that in Italy the healthcare system is essentially public and entirely tax funded. Consequently, until 2017 the management of hospitals’ medical liability was entirely assigned to private insurance companies that evaluate and compensate patients’ damage claims [45]. In recent years the costs of hospital insurance have skyrocketed, with the result that only a few companies, mainly foreign, were able to cover the medical malpractice risks. 

In this situation, the Law n° 24 of March 8, 2017, represents a cornerstone that states the need to integrate patient safety and risk management from clinical, economic, and legal standpoints [46,47]. Next, the matter of medical liability has been dealt with in this law, through introducing provisions regarding penal and civil consequences of medical errors, the need for healthcare providers to follow only guidelines validated by the Ministry of Health, and the role of private and public hospitals in the management of litigation and malpractice claims and lawsuits. 

On the basis of this complex law, the role of legal medicine experts has been confirmed in hospital clinical RMU, even in evaluation of economic costs of medical malpractice victims. In Italy, university medico-legal training offers students the opportunity to become experts in forensic pathology as well as in evaluation of damages in personal injury cases. Insurance companies generally pay medico-legal consultants for estimating damage claims as a consequence of medical malpractice. Hospital RMU have a multidisciplinary competence; they are directly connected with the local Legal Office, and one of their fundamental objectives is to prevent and to compensate organizations and healthcare providers in case of adverse events and errors [48]. In Italy, patients or family members who demand compensation for medical injury assert that a negligent act or omission caused the injury. The Legal Office directs the medico-legal expert of the RMU to visit the patient if still alive and analyze all his/her medical records for providing an initial case assessment. The hospital must prove that no harm was caused to the patient and/or that the adverse event was not avoidable with diligent/prudent actions by healthcare professionals. The clinical RMU involves the doctors/nurses who delivered care to the claimant and verifies whether the medical/hospital standards/care pathways were met. 

The aim of this medico-legal analysis is twofold. Firstly, by examining the causes of the adverse event/error it is possible to map and validate the care pathway processes, and implementation of all the tools and proactive measures to prevent it. In this phase, it is possible to assess if and what kind of guidelines and evidence-based practices were used. If an avoidable error was identified, the RMU communicates to the Legal Office the need to compensate the injury and the value of the patient’s damage. A Claims Management Committee (CMC) nominated by the hospital governance provides that the damages be compensated directly by the hospital, removing the cost of liability insurance, and guaranteeing greater diligence and efficiency with lower economic outlay [49]. According to Studdert et al., who reviewed a random sample of malpractice claims from five liability insurers in the U.S., claims not involving errors accounted for 13 to 16% of the system’s total monetary costs [50]. On the other hand, Italy has the highest proportion of health lawsuits settled in court in Europe and is accordingly in last place concerning health lawsuits settled out of court. The Italian judiciary has thus become the greatest defender of the health of patients against medical malpractice [51]. Hence, the CMC establishes the cost of the claim and tries to pursue a hospital for compensation avoiding a lawsuit.

## 4. Medical Liability Claims for Nosocomial Infections: Medico-Legal Perspectives

Sepsis related to HAI is one of the serious adverse events in healthcare. The most common types of infections may be caused by bacteria, viruses, fungi, and parasites and develop from surgical procedures, catheters in the urinary tract, blood vessels, or material inhaled into the lungs. HAI cause a significant burden for hospitalized patients in terms of length of mortality, morbidity, length of stay, and costs [4]. From a medico-legal perspective, NI represent a challenge as regards correct diagnosis which is very difficult to accomplish even by linking the medical records with the autopsy and related postmortem laboratory findings. The medico-legal expert working in the hospital RMU together with a multidisciplinary group must monitor and implement the organization measures to prevent NI. Moreover, claims for medical liability involving NI require a complete case analysis, evaluating the possibility of compensating the patient or deciding to defend the hospital in lawsuits settled in court. 

Prevention of NI is the responsibility of all individuals and services providing healthcare. Therefore, hospital administrations appoint an Infection Control Committee (CIO) who is responsible for establishing and maintaining infection prevention and control, its monitoring, surveillance, reporting, research, and education. The CIO includes wide representation from all relevant disciplines and departments. 

The hospital pharmacists together with microbiologists and infectious disease experts play a fundamental role in management of NI. They are responsible not only for storing and dispensing the best evidenced anti-infectious drugs but also in development of pathways/practices for everyday sterilization and disinfection in hospital wards [52]. It has been hypothesized that involvement of clinical pharmacists in care of patients with HAI could be associated with improved clinical and economic outcomes [53]. Determination of injuries or death caused by sepsis or septic shock associated with HAI requires an analysis of the specific circumstances of how the infection was acquired, if it was appropriately diagnosed and treated, and whether it could have been prevented. 

Adhering to very stringent and standardized HAI preventive measures to minimize infections has a fundamental defensive value in management of medical liability claims. Conversely, failure to follow safe practices can amount to medical negligence [54,55]. In such a case the patient/plaintiff must prove the injury correlated to infection, while the hospital needs to demonstrate its adoption of the best standards for organizational and iatrogenic infections risk factors. These include cleanliness of the wards, concentration of patient beds, sterility of medical devices and surgery rooms. Iatrogenic risk factors mainly relate to the care providers’ practices of hygiene, use of antibiotics, and the degree of care during invasive procedures [56,57]. 

Actually, the medico-legal expert evaluates all the medical records dealing with the patients’ diseases as well as the certified steps of hospital ward sterility and infection control measures. It is also important to disclose information to patients and family members. Many times, infections and sepsis are not preventable, and even if well treated, they can progress until death; it depends on the assessment of the patient’s disease severity. Hence, there is a fundamental role of the information given to the patient and relatives during hospitalization, regarding the likelihood of having an infection strictly not linked to negligence in prevention and control of hospital microorganisms but only with the severity of the disease. With regard to NI, medico-legal analysis of the complaint or claim for compensation allows to highlight the need to introduce or improve best-practices to prevent infections. This means also reporting negligent acts of providers or the organizational lack of tools or measures to preserve patient safety. Moreover, a medico-legal report in such a case is useful for arriving at the CMC verdict for compensating the plaintiff or not. 

In an era in which public hospitals have a great economic burden for sustaining average/high standards of care, it is sometimes difficult to prove that every HAI control and preventive measure has been carried out. It could be enough to demonstrate the absence of qualified procedures for hand hygiene or surgery room sterilization to sustain a claim of negligence on the part of the hospital administrators or staff. Therefore, the medico-legal expert’s task in such cases is multidisciplinary, but regarding malpractice claims, the expert has to evaluate: (i) The correct diagnosis of NI; (ii) the diligent or negligent management by the organization/providers; (iii) whether the damage claimed by the patient is due to infections or to severity of patient’s pre-existing disease. 

In cases where an error has occurred or there is lack of evidence of best-practice use, the policy is to compensate it out of court. Generally speaking, the awards after a lawsuit on average could be triple or fourfold the size of compensation made directly by the hospital. Compensation and apology for an error is a way also to restore the trust among patients that represents the cornerstone of the doctor‒patient relationship and the core of the people’s perception of the institution [41].

## 5. Conclusions

Despite the fact that sepsis remains a leading cause of morbidity and mortality, affecting over 30 million people worldwide each year, public awareness is poor and associated NI are still difficult to diagnose [58,59,60,61,62]. Medico-legal experts in Italy face sepsis and hospital infections in at least three phases. As forensic pathologists, they have the challenging task of diagnosing sepsis and its etiology through autopsy and ancillary postmortem examinations. The growing interest in patient safety and in reducing the economic costs of healthcare have led to an enhancement in the status of medico-legal experts working in hospital Clinical Risk Management Unit. Their role in risk assessment and improving best practices and care pathways is fundamental for guaranteeing patients’ safety. Moreover, in medical malpractice claims the medico-legal expertise’s acquaintance with legal issues and role in damage assessment allow the hospital administration to do the best regarding the plaintiff’s compensation claim, never forgetting to preserve the trust with patients and their kin. The challenging diagnosis and the difficulty in prevention, control, and compensation for NI give medico-legal providers a broad area of expertise, wherein only with continuing professional training will they be able to cope with healthcare system needs. 
